# Integrated transcriptomic and BSA-seq analyses identify novel QTL and candidate genes for stay-green in maize

**DOI:** 10.3389/fpls.2026.1936883

**Published:** 2026-09-24

**Authors:** Ran Zheng, Dan Lv, Kunyi Shen, Qinggui Wu, Hongbing Luo, Tao Chang

**Affiliations:** 1 Hunan Medical College, Huaihua, China; 2 Department of Biology and Food Engineering, Lyuliang University, Lvliang, China; 3 Hunan Agricultural University, Changsha, China

**Keywords:** leaf senescence, maize, QTLs, RNA-Seq, stay-green

## Abstract

**Introduction:**

The stay-green trait is crucial for enhancing maize yield and stress tolerance, yet its genetic basis in new germplasm remains underexplored. This study aimed to dissect the molecular mechanisms underlying stay-green in the inbred line T01 using an integrated approach.

**Methods:**

We constructed an F₂:₃ population from T01 and the non-stay-green line Mo17, and combined BSA-seq to map candidate loci, RNA-seq at three developmental stages to profile transcriptomic dynamics, and WGCNA to identify co-expression networks.

**Results:**

BSA-seq mapped two candidate QTL regions on chromosomes 5 and 9, with the former representing a potentially novel locus. Transcriptomic analysis revealed stage-specific regulation, with enrichment in reactive oxygen species metabolism, photosynthesis, and chloroplast maintenance at 10, 20, and 30 days after pollination (DAP), respectively. WGCNA identified six hub genes functionally involved in transcriptional regulation, ROS scavenging, and photosynthesis. Notably, three genes (*ZmFSD3*, *ZmWRKY62*, and *ZmNAC29*) were located within BSA-defined intervals and supported by WGCNA analysis, with their expression patterns validated by RT-qPCR.

**Discussion:**

Our findings reveal a complex, multi-pathway regulatory network in which WRKY and NAC transcription factors are highly connected candidate nodes. This provides new genetic resources and a theoretical basis for molecular breeding of stay-green maize.

## 1 Introduction

Maize (*Zea mays* L.) is one of the most important cereal crops worldwide, and stay-green trait is a critical determinant of grain yield and stress tolerance (Kamal et al., 2019; Chibane et al., 2024). Stay-green maize genotypes can maintain leaf greenness and photosynthetic activity during the grain-filling stage, thereby extending the duration of grain filling, enhancing dry matter accumulation, and ultimately improving both grain yield and stalk quality (Chen et al., 2013; Gao et al., 2023). Under abiotic stress conditions such as drought and high temperature, the stay-green trait plays an especially important role in ensuring stable yield production (Celine, 2019; Gao et al., 2022; Kumar et al., 2022). Therefore, dissecting the molecular and genetic mechanisms underlying stay-green trait and identifying key genes associated with stay-green are of great theoretical and practical significance for breeding new maize varieties with high yield, superior quality, and enhanced stress resilience.

Although QTL for stay-green have been identified on multiple maize chromosomes (Almeida et al., 2014), and transcription factors including *NAC7* (Yuan et al., 2023) and *WRKY* (Li et al., 2024) members are known to regulate leaf senescence, the overall regulatory network controlling this trait has not been fully characterized. The resolution of conventional QTL mapping is relatively low, and the majority of studies have relied on a single strategy, such as QTL analysis or transcriptome profiling, leaving a gap between genetic loci, dynamic expression changes, and the underlying regulatory network.

In our previous study (Zheng et al., 2023), BSA-seq and RNA-seq were used to preliminarily characterize the stay-green inbred line T01, but co-expression network analyses such as WGCNA were not performed. Given that regulatory mechanisms may differ across genetic backgrounds and that most studies have focused on a limited set of accessions, the exploration of stay-green-related genes in new germplasm remains limited, and the specific genes and networks governing this trait in T01 are largely unknown. Here, we integrated BSA-seq, RNA-seq, and WGCNA to identify candidate genes potentially involved in regulating maize stay-green, with the goal of addressing these gaps and elucidating the molecular basis of stay-green in this new material.

To address these issues, we used the stay-green inbred line T01 and the non-stay-green inbred line Mo17 as materials. We constructed an F2:3 population, and combined BSA-seq to rapidly map candidate regions associated with the stay-green trait, RNA-seq to compare gene expression differences at different growth stages, and WGCNA to identify co-expression modules and hub genes highly correlated with stay-green. This network-based approach enables us to reveal the hierarchical regulatory network, identify hub genes as central nodes, and cross-validate candidates by intersecting BSA-mapped QTL intervals with WGCNA-derived hub genes. Our objectives were first to identify new QTL intervals associated with stay-green in maize, second to screen key candidate hub genes for the stay-green trait, and third to decipher the molecular regulatory network underlying stay-green. The results will provide new gene resources and a theoretical basis for molecular breeding of stay-green in maize, and will also serve as a reference for the application of combined BSA-seq and RNA-seq strategies in the study of complex traits.

## 2 Results

### 2.1 Phenotypic analysis of stay-green differences between two maize genotypes

Phenotypic differences between the two genotypes are shown in **Figure 1**. At 10 DAP, the two lines showed little difference in leaf color (**Figure 1A**). At 20 DAP, Mo17 leaves began to show yellowing and senescence symptoms, while T01 remained green. By 30 DAP, Mo17 leaves were mostly withered, whereas T01 was just beginning to yellow. At 40 DAP, Mo17 plants had turned completely yellow, but T01 still maintained considerable green tissues (**Figure 1B**).

**Figure 1:**
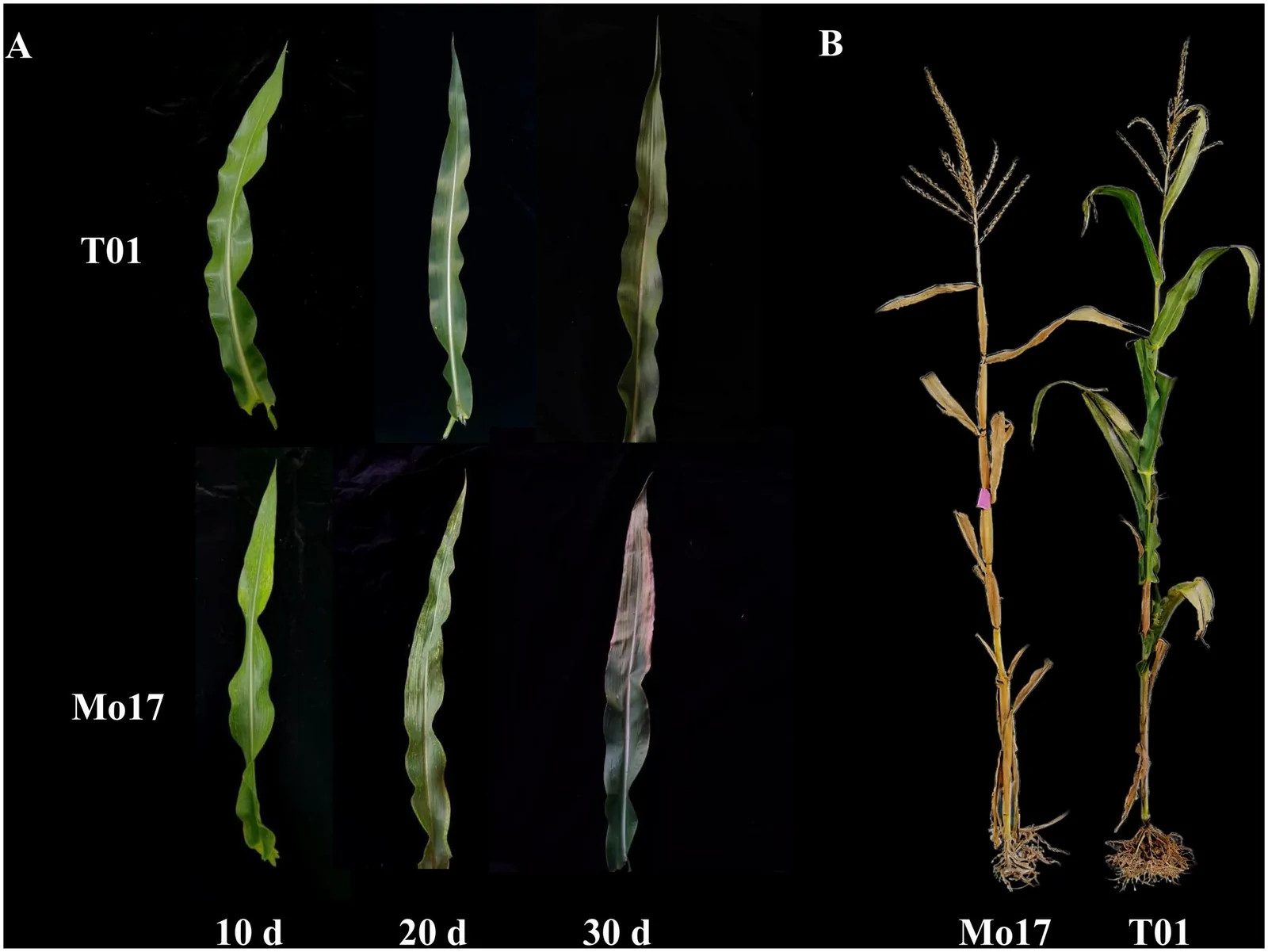
Phenotypic differences in stay-green between T01 and Mo17. **(A)** Leaf color of the two genotypes at 10, 20, and 30 DAP. **(B)** Whole-plant phenotype of the two genotypes at 40 DAP.

The stay-green-related traits of T01 and Mo17 are compared in **Supplementary Figure S1**. Overall, T01 outperformed Mo17 across most parameters, particularly in photosynthetic efficiency and biomass accumulation. Specifically, T01 exhibited significantly higher visual stay-green, chlorophyll content, and net photosynthetic rate (Pn) than Mo17 at all three time points, with the differences becoming more pronounced by 30 days, indicating a superior stay-green capacity and photosynthetic performance in T01. In contrast, Mo17 consistently maintained higher intercellular CO_2_ concentration (Ci) than T01 throughout the experimental period. Regarding growth traits, T01 showed significantly greater biomass than Mo17 at 20 and 30 days, whereas the 100-grain weight did not differ significantly between the two genotypes at 10 days, though Mo17 exhibited a slight advantage at 20 and 30 days. Collectively, these results demonstrate that T01 possesses a distinct advantage in photosynthetic efficiency and overall biomass accumulation.

Overall, the two genotypes began to diverge phenotypically at 20 DAP, with T01 showing a much stronger stay-green trait than Mo17.

### 2.2 Cellular analysis of two maize genotypes

Transmission electron microscopy was used to examine chloroplast ultrastructure in leaf cells of the two materials at different developmental stages (**Figure 2**). At 10 DAP, no obvious structural differences were observed between the two genotypes (**Figures 2A, B**). By 20 DAP, chloroplasts in Mo17 appeared nearly disrupted; compared with T01, Mo17 exhibited fewer grana and a greater accumulation of osmiophilic globules (**Figures 2C, D**). At 30 DAP, grana number began to decline in T01, whereas in Mo17, chloroplasts were almost completely disintegrated, accompanied by a further increase in osmiophilic globules (**Figures 2E, F**). Quantitative analysis of chloroplast morphology revealed that T01 consistently maintained larger chloroplast area and higher grana density than Mo17 across all stages, with the most pronounced differences observed at 20 and 30 DAP (**Supplementary Table S1**).

**Figure 2:**
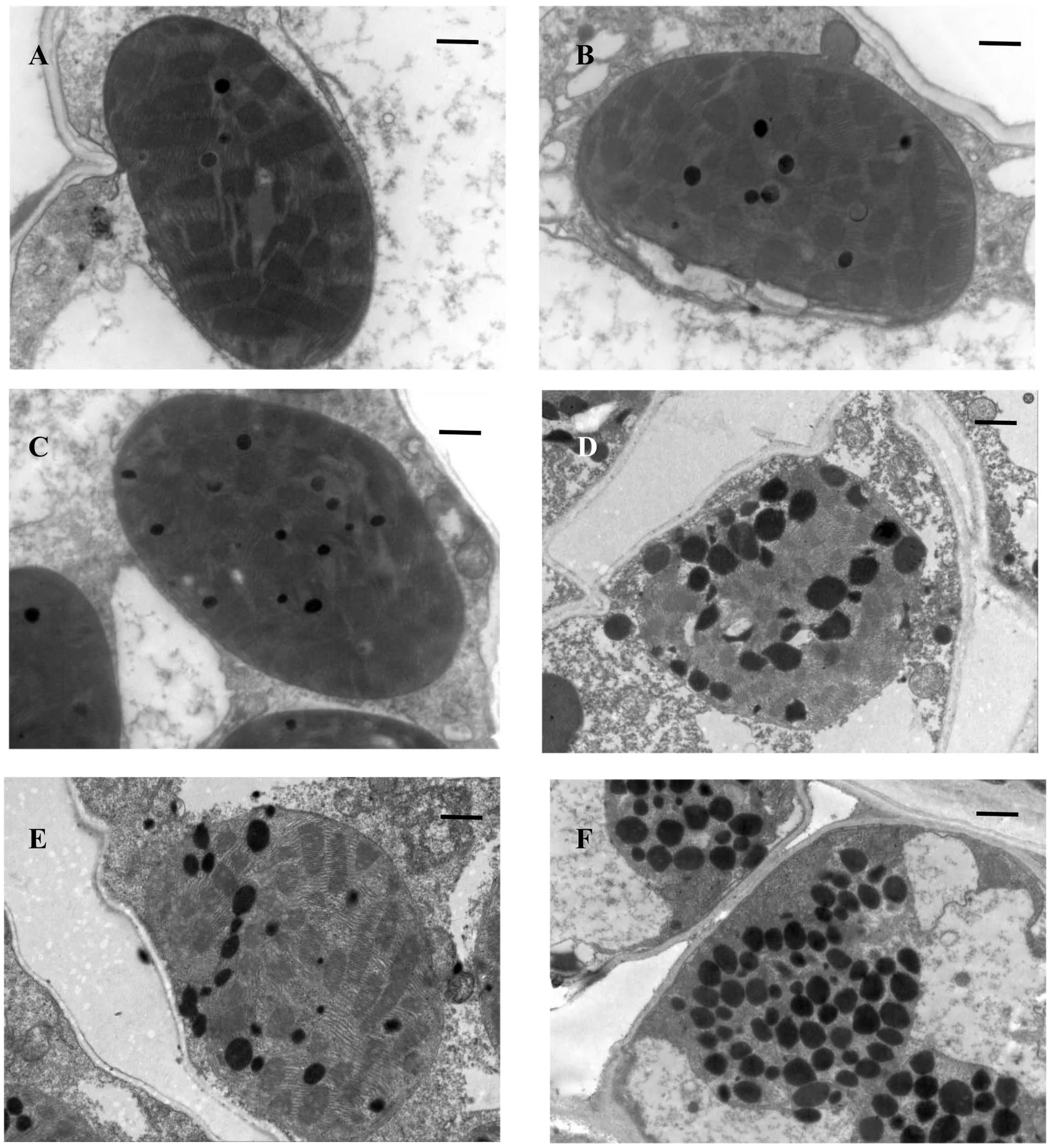
Microscopic phenotypic differences between leaves of T01 and Mo17. **(A)** T01 at 10 DAP. **(B)** Mo17 at 10 DAP. **(C)** T01 at 20 DAP. **(D)** Mo17 at 20 DAP. **(E)** T01 at 30 DAP. **(F)** Mo17 at 30 DAP. Scale bars = 0.2 μm.

### 2.3 BSA and mapping of stay-green candidate genes

We performed resequencing of seven DNA libraries, comprising the two parental lines and four extreme bulks, on the MGISEQ-2000 platform, yielding 704.6 Gb of high-quality data. Specific sequencing parameters and quality control data are shown in **Supplementary Tables S2**, **S3**. By intersecting the initial mapping results from seven algorithms, we identified two chromosomal regions associated with the stay-green trait (**Supplementary Table S4**). Specifically, a region was retained only if it was consistently detected by all seven algorithms, and for each algorithm the 95% confidence threshold was determined by 1,000 permutation tests. The overlapping consensus intervals were then defined as the final candidate regions. The distribution of SNP/InDel markers across the two candidate intervals on chromosomes 5 and 9 was visualized (**Figure 3A**), and the ΔSNP-index values of these intervals were plotted to highlight the significantly associated genomic regions (**Figure 3B**). To confirm these findings, we screened 445 InDel markers between the two parents and obtained 38 polymorphic markers (**Supplementary Table S5**). Among these, six markers exhibited polymorphism in the extreme bulks (**Figure 3C**), including C5-7, C5-8, 5In9, and 5In12 on chromosome 5, as well as C9–5 and C9–11 on chromosome 9. Within the two candidate intervals, we identified 13 and 21 annotated genes within the chromosome 5 and 9 intervals, respectively (**Supplementary Tables S6**, **S7**).

**Figure 3:**
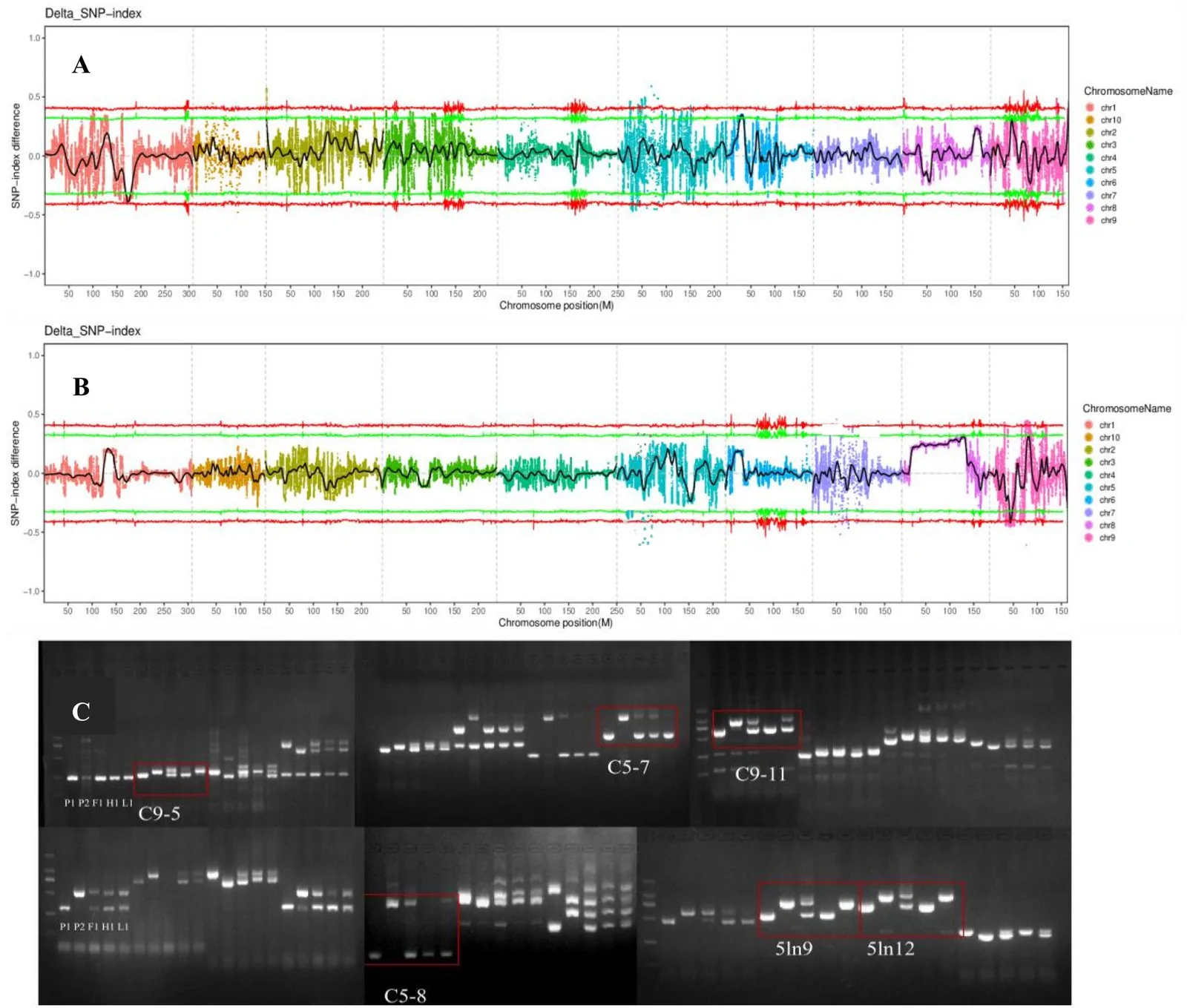
BSA-based mapping of stay-green genes. **(A)** Distribution of SNP/InDel markers in the candidate intervals on chromosomes 5 and 9. **(B)** ΔSNP-index values of the candidate intervals. **(C)** Validation of polymorphic InDel markers among the parents and extreme bulks.

### 2.4 Transcriptome analysis of two maize genotypes

To dissect the molecular mechanisms underlying the difference in stay-green trait between T01 and Mo17, we performed transcriptome sequencing on leaves collected at three developmental stages. The specific sequencing results are shown in **Supplementary Table S8**. Principal component analysis (**Figure 4A**) showed that the first two principal components accounted for 29.29% and 16.63% of the total variance, respectively, indicating that the data were of sufficient quality for subsequent analyses. In pairwise comparisons between the two inbred lines at the same developmental stage, we identified 71 upregulated differentially expressed genes (DEGs) common to all three stages (**Figure 4B**) and 21 downregulated DEGs common to all three stages (**Figure 4C**). Specifically, at 10 DAP, 20 DAP, and 30 DAP, we identified 4563, 4560, and 8157 downregulated DEGs, and 3895, 3863, and 4600 upregulated DEGs, respectively. GO enrichment analysis (**Figure 4D**) revealed that the DEGs at the three stages were functionally classified into biological process, cellular component, and molecular function categories. At 10 DAP, DEGs were mainly enriched in terms related to reactive oxygen species response, plastid, and nuclease activity. At 20 DAP, DEGs were predominantly enriched in photosystem I light harvesting, chloroplast thylakoid membrane, and oxidoreductase activity. At 30 DAP, DEGs were primarily enriched in chloroplast organization, chloroplast, and catalytic activity. Given that chloroplast development and redox-related processes were implicated at all three stages, these two biological processes may be closely associated with the regulation of stay-green trait, consistent with functional roles previously reported for senescence-related genes in maize and other species (Mao et al., 2017; Sekhon et al., 2019; Gao et al., 2023). KEGG pathway enrichment analysis (**Figure 4E**) showed that the photosynthetic antenna proteins pathway was significantly enriched across all three stages. Additionally, glutathione metabolism was commonly enriched at 10 and 20 DAP, while zeatin biosynthesis and peroxisome were co-enriched at 20 and 30 DAP, suggesting that these pathways may play important roles in the regulation of stay-green trait.

**Figure 4:**
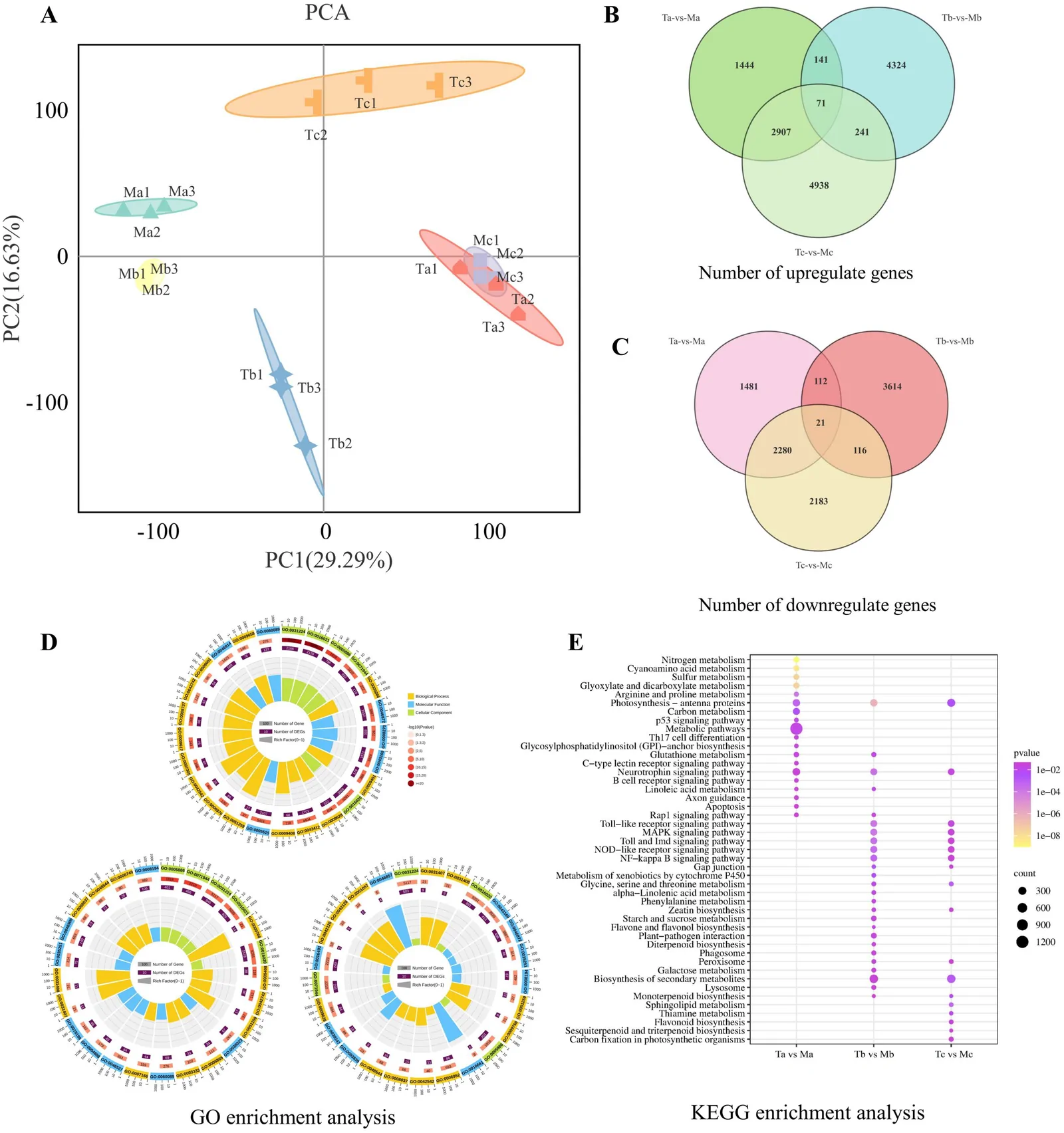
Comparative transcriptome analysis of two maize genotypes. **(A)** PCA of transcriptome. **(B)** Upregulated of DEGs between the two materials. **(C)** Downregulated DEGs between the two materials. **(D)** GO enrichment analysis. **(E)** KEGG enrichment analysis.

### 2.5 WGCNA identifies key candidate genes associated with the stay-green trait

To identify gene modules highly associated with the stay-green trait, we performed WGCNA on genes with expression variance ranking in the top 50% across all samples from T01 and Mo17 at different pollination stages (**Figure 5**). A soft-thresholding power of β=30 (**Supplementary Figure S2**) was selected based on the scale-free topology criterion, ensuring that the constructed network approximates a scale-free topology. A total of 24 significantly associated co-expression modules were obtained, each represented by a distinct color, with the gray module considered invalid (**Figure 5A**). The brown2 module contained the largest number of genes (2363), followed by brown (2021), antiquewhite2 (1408), darkolivegreen1 (1349), and coral3 (1211), while the deeppink module contained the fewest (37). Detailed module composition and gene counts are provided in **Supplementary Table S9**. Based on module-trait correlation coefficients (**Figure 5B**), we identified 11 modules significantly associated with the visual stay-green (VSG) trait, among which six showed significant positive correlations and five showed significant negative correlations. KEGG enrichment analysis of genes within the two modules most highly correlated with the stay-green trait (|r| > 0.90, *P* < 0.01), namely darkolivegreen1 and brown2, revealed that genes in these modules were mainly involved in pathways such as photosynthesis, peroxisome, plant hormone signal transduction, and starch and sucrose metabolism (**Figure 5C**). To further screen for core candidate genes, we calculated module membership (kME) and gene significance (GS) for each gene within these two modules (**Supplementary Table S10**). The top 10 genes with the highest kME values within each module were retained as hub genes (**Figure 5D**). Through functional annotation and literature comparison, we ultimately selected four highly connected genes from the darkolivegreen1 module: *Zm00001eb057820*, *Zm00001eb106810*, *Zm00001eb226600*, and *Zm00001eb358500*, and two highly connected genes from the brown2 module: *Zm00001eb395130* and *Zm00001eb395320*. Integrating these results with the BSA analysis, three genes, *Zm00001eb226600* located at Bin 5.03, and *Zm00001eb395130* and *Zm00001eb395320* located at Bin 9.05, were detected by both approaches. Functional annotation assigned these three genes as *ZmFSD3*, *ZmWRKY62*, and *ZmNAC29*, respectively, which are used throughout the remainder of this study.

**Figure 5:**
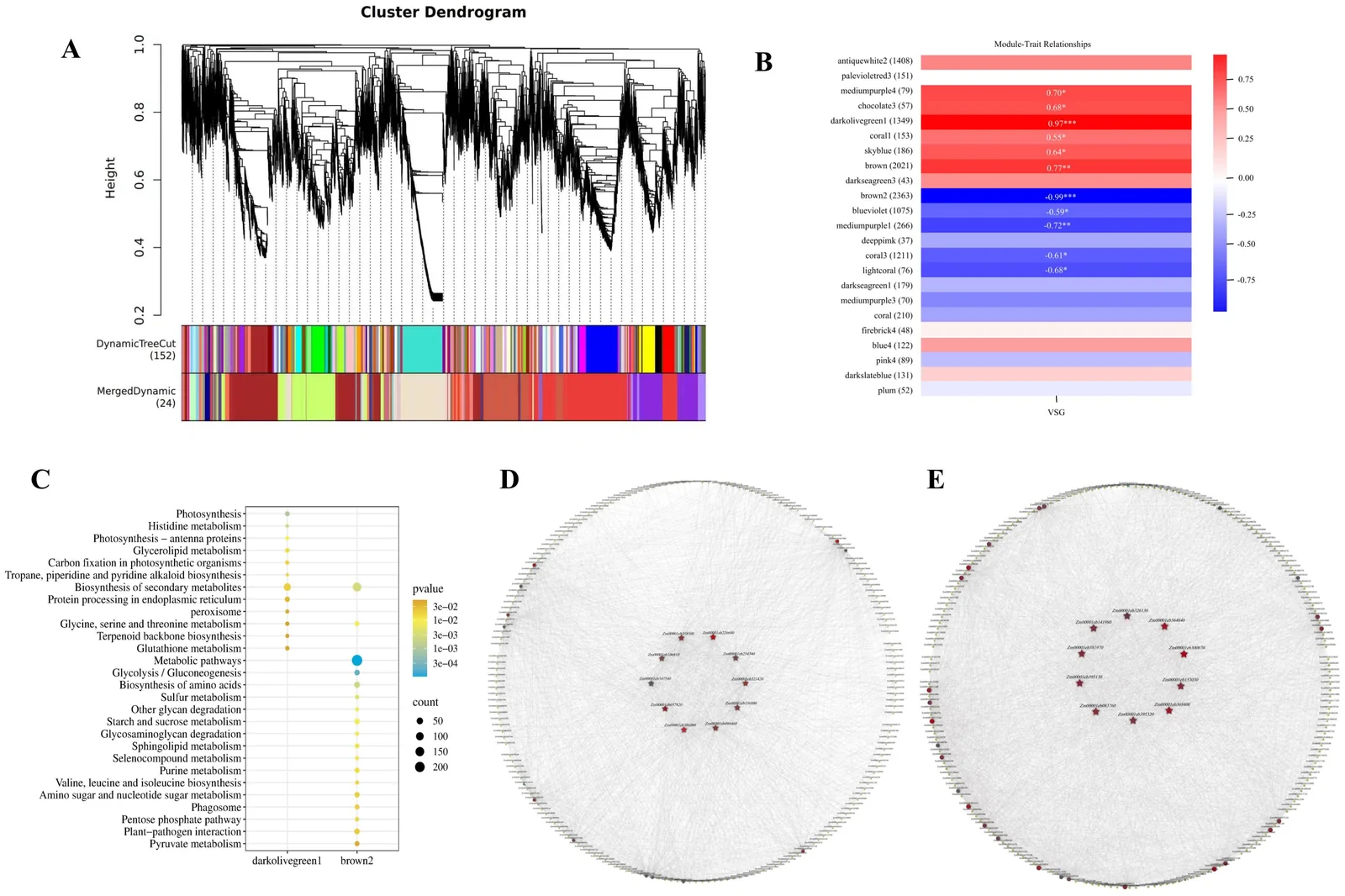
Screening and identification of hub genes by WGCNA. **(A)** Dendrogram of co-expression modules identified by WGCNA. **(B)** Heatmap of correlations between modules and the stay-green trait. **(C)** KEGG enrichment analysis of the darkolivegreen 1 module. **(D)** KEGG enrichment analysis of the brown 2 module. **(E)** Co-expression network of the darkolivegreen 1 module showing hub genes. **(F)** Co-expression network of the brown 2 module showing hub genes.

### 2.6 Expression validation and patterns of candidate genes

We performed RT-qPCR analysis on the three candidate genes described above, and the results are presented in **Figure 6**. *ZmFSD3* showed high expression in T01 at the middle and late stages. In contrast, *ZmWRKY62* and *ZmNAC29* exhibited low expression in T01 across all three stages. These expression patterns have a linear fit value higher than 0.99 with transcriptome data (**Supplementary Figure S3**), indicating a consistent trend. Indicating that these genes may be candidate genes involved in maize stay-green traits.

**Figure 6:**
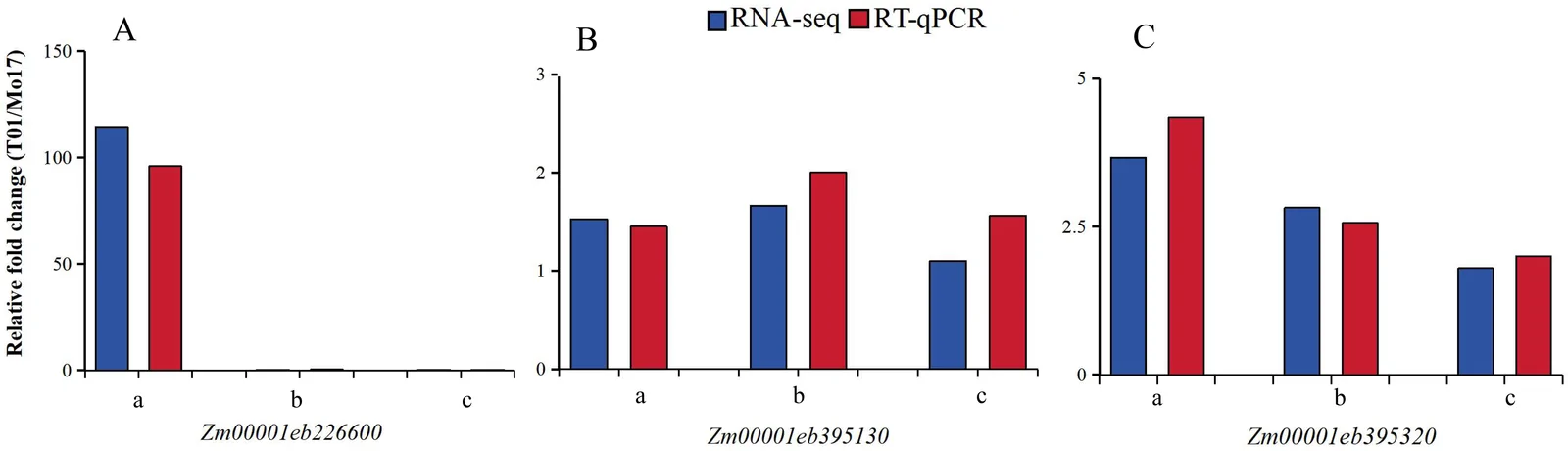
FPKM and RT-qPCR of the expression of three hub genes in T01 and Mo17 at three stages. The values represent the relative expression fold change of Mo17/T01. **(A–C)** represent the fold changes of Zm00001eb226600, Zm00001eb395130, and Zm00001eb395320, respectively. a, b, and c represent 10, 20, and 30 DAP, respectively.

## 3 Discussion

In this study, we integrated BSA-seq, RNA-seq, and WGCNA to systematically dissect the stay-green inbred line T01, the non-stay-green line Mo17, and their derived F2:3 population in maize. We explored the molecular basis of stay-green trait from three perspectives: genetic loci, expression dynamics, and regulatory networks. Compared with studies relying on a single strategy, our multi-level joint analysis strategy, which integrates information on genetic mapping, temporal expression patterns, and network hub nodes, offers a useful reference for dissecting complex quantitative traits. In contrast to our previous work on the same T01×Mo17 population (Zheng et al., 2023), which focused primarily on lists of differentially expressed genes, the present study used WGCNA to construct a co-expression network and identify hub genes, providing new insights into the regulatory architecture of stay-green. In addition, three of these hub genes were located within the QTL intervals mapped by BSA-seq, providing cross-validation from two independent approaches.

### 3.1 BSA-seq revealed the germplasm specificity and genetic complexity of stay-green QTLs

Through BSA-seq in the T01 × Mo17 population, we mapped two candidate intervals associated with the stay-green trait, located on chromosome 5 (56.15–57.09 Mb) and chromosome 9 (137.05–138.24 Mb). The interval on chromosome 9 partially overlapped with a stay-green-related QTL reported by Zheng et al. (2023) using the same parental combination, suggesting that this region is repeatably detectable across different mapping methods and population generations and may harbor key genes governing stay-green regulation. The interval on chromosome 5 from 56.15 to 57.09 Mb has rarely been reported in previous stay-green QTL studies in maize. A comparison with previously mapped stay-green QTL intervals (Almeida et al., 2014; Kante et al., 2016) showed that no reported loci overlap this region, suggesting that T01 may carry genetic variants distinct from those in other stay-green germplasms. Similarly, Zhang et al. (2019) identified a novel stay-green QTL in a mapping population derived from IHP1 and ILP1, further indicating that the genetic architecture of stay-green may vary across different backgrounds. As a complex quantitative trait controlled by multiple genes, stay-green may be achieved through different molecular strategies in different germplasms to accomplish the biological goal of delayed senescence. The detection of a T01-specific QTL on chromosome 5 enriches the diversity map of stay-green genetic loci in maize and provides an alternative target region for future cloning of novel functional genes. Nevertheless, due to limited recombination events within this interval in the F2:3 population used in this study, further fine-mapping through expanded population size or development of high-density markers within the interval will be required to narrow down the list of candidate genes. Fine-mapping with larger populations is needed to further validate these intervals.

### 3.2 Transcriptome analysis revealed that stay-green depends on stage-specific synergistic regulation across multiple pathways

Through comparative transcriptomic analysis of T01 and Mo17 at three time points after pollination, we observed that the biological functions of differentially expressed genes exhibited a clear temporal shift. At 10 DAP, the enriched terms were predominantly related to reactive oxygen species metabolism and redox processes. At 20 DAP, the enriched functions gradually shifted toward photosynthetic light harvesting and thylakoid membrane components. At 30 DAP, the enrichment was mainly focused on chloroplast structure maintenance and catalytic activity. This dynamic pattern suggests that the stay-green phenotype of T01 does not rely on continuous high-level activation of a single defense pathway. Instead, T01 appears to activate different functional modules sequentially at distinct senescence stages, and these modules are enriched in pathways related to stage-specific chloroplast protection. More specifically, the early-stage DEGs were enriched in antioxidative pathways, the mid-stage DEGs in photosystem components, and the late-stage DEGs in chloroplast structural maintenance, suggesting stage-specific functional shifts during senescence. However, these inferences are derived from correlational expression data and require functional validation. In terms of KEGG pathways, photosynthetic antenna proteins were significantly enriched across all three stages, whereas glutathione metabolism was enriched only at the early stage, and zeatin biosynthesis and peroxisome were enriched at the middle and late stages. This time-specific distribution further supports the stage-specific regulatory hypothesis. These findings are generally consistent with the preliminary transcriptomic observations of T01 stay-green reported by Zheng et al. (2023), while the systematic comparison across three time points in this study provides a more complete depiction of the temporal dynamics underlying this regulatory process. The enrichment of these pathways across developmental stages is further supported by functional studies linking ROS scavenging, photosynthetic maintenance, and chloroplast integrity to delayed leaf senescence (Mao et al., 2017; Sekhon et al., 2019; Gao et al., 2023).

### 3.3 WGCNA revealed the multi-level integration mechanism of the stay-green regulatory network

WGCNA identified six hub genes with high connectivity from the darkolivegreen 1 and brown 2 modules, which were most highly correlated with the stay-green trait. These genes were functionally annotated to multiple layers, including transcriptional regulation represented by 3 genes, photosynthetic light harvesting through a chlorophyll a-b binding protein, reactive oxygen species scavenging via *ZmFSD3*, and chlorophyll biosynthesis involving uroporphyrinogen decarboxylase (Chai et al., 2019; Sekhon et al., 2019; Wang et al., 2024). This functional diversity suggests that the molecular basis of stay-green is a complex regulatory network with multiple nodes and pathways, rather than being governed by a single major gene. However, as BSA-seq is primarily a qualitative mapping strategy, it does not allow direct estimation of the phenotypic variance explained by individual QTLs. Further fine-mapping using larger segregating populations will be required to precisely evaluate their genetic effects. Of particular note, *ZmWRKY57* is homologous to known senescence repressors *WRKY54* and *WRKY70* in Arabidopsis, while *ZmWRKY62* encodes a *WRKY62* protein and is phylogenetically related to the senescence activator *WRKY45*. Chen et al. (2017) demonstrated that *WRKY45* can directly bind to the promoters of senescence-associated genes and activate their expression. To date, however, functional characterization of these maize WRKY homologs in relation to stay-green has been lacking. Drawing on the established roles of their Arabidopsis counterparts, we speculate that the opposing expression trends of these two *WRKY* genes between T01 and Mo17, one potentially inhibitory and the other activating, may represent an antagonistic regulatory module, and that the relative abundance of their transcripts could influence the timing of senescence onset. Furthermore, *ZmNAC29*, a member of the *NAC* transcription factor family, shares homology with *OsNAC2* in rice, which has been shown to directly activate the expression of chlorophyll degradation genes *OsSGR* and *OsNYC3* (Mao et al., 2017), raising the possibility that *ZmNAC29* may fulfill a comparable function in maize. Given that no direct evidence is yet available for this gene in the context of maize stay-green, this hypothesis awaits validation through future targeted experiments. However, the specific function of *NAC29* in maize stay-green has not been previously reported. We therefore prioritized this gene as a candidate. Beyond the candidate genes themselves, the network architecture revealed here raises broader questions about how transcriptional regulators of the *WRKY* and *NAC* families coordinate with metabolic pathways to modulate senescence timing—an issue that can be progressively dissected by combining the candidate loci identified here with targeted mutagenesis and biochemical assays. The persistent significance of reactive oxygen species-related pathways in the transcriptome enrichment analysis was corroborated by the identification of *ZmFSD3* as a hub gene. The homologous gene of *FSD3* in *Arabidopsis*, *AT5G23310*, encodes an iron superoxide dismutase involved in reactive oxygen species scavenging and redox signaling regulation. This cross-validation further supports the importance of redox homeostasis in delaying senescence. Integrating these findings with the BSA mapping results, *ZmFSD3* located at Bin 5.03, along with *ZmWRKY62* and *ZmNAC29* located at Bin 9.05, were consistently detected by both genetic mapping and co-expression network analysis. This cross-validation enhances the confidence in these genes as credible candidates. RT-qPCR validation demonstrated that their expression trends were consistent with the transcriptome data, confirming the reliability of the expression profiles. Beyond the candidate genes themselves, the multi-omics integration strategy employed here exemplifies a powerful approach to dissect complex quantitative traits. Similar integrative frameworks, combining transcriptomic profiling with genetic analyses, have proven effective in identifying key regulatory nodes governing stress-responsive pathways in other crop species (Liu et al., 2026). Such cross-species methodological parallels underscore the broader applicability of our integrated BSA-seq, RNA-seq, and WGCNA pipeline for elucidating the genetic architecture of agronomically important traits.

In summary, through the integrated analysis of BSA-seq, RNA-seq, and WGCNA, this study identified germplasm-specific QTLs associated with the stay-green trait, revealed that stay-green relies on a stage-coordinated and multi-pathway defensive strategy, and constructed a co-expression regulatory network in which WRKY and NAC transcription factors stand out as candidate hub regulators. These findings provide new genetic resources and theoretical foundations for molecular breeding of stay-green in maize, and also offer a reference for the application of combined BSA-seq and RNA-seq strategies in dissecting complex traits. Functional validation of the hub genes identified in this study will be pursued through gene editing technologies in future work, with the aim of further testing the proposed regulatory network hypothesis and evaluating the practical utility of these candidate genes in breeding programs.

## 4 Materials and methods

### 4.1 Materials

The maize stay-green inbred line T01 was developed by College of Agronomy, Hunan Agricultural University, while the non-stay-green inbred line Mo17 is a publicly available reference genome line. An F1 population was generated by crossing T01 (as male parent) with Mo17 (as female parent), and the F1 plants were self-pollinated to produce the F2 population. Subsequently, F2:3 families were established by selfing individual F2 plants and used for BSA-seq and phenotypic evaluation. All experimental materials were grown at the Yunyuan Experimental Station in Changsha, Hunan Province (113.42°E, 34.48°N) and the Liuyang Experimental Station (109.20°E, 18.40°N), under standard field management practices.

### 4.2 Phenotypic analysis

#### 4.2.1 Leaf stay-green evaluation

At 10(stage a), 20(stage b), and 30(stage c) DAP, leaf color changes and overall plant stay-green performance were observed and recorded for both T01 and Mo17. Ten representative plants per line were selected and photographed. Leaf samples were also collected at the corresponding time points for subsequent cytological observation and RNA extraction.

Phenotypic evaluation was performed at 10, 20, and 30 DAP. For each genotype and time point, ten representative plants were photographed for whole-plant documentation. To quantify the stay-green phenotype, all green leaves from each plant were collected, and the area of each leaf was manually measured. The total green leaf area per plant was summed, and the VSG value was calculated as the percentage of total green leaf area retained relative to that at 0 DAP, following the method of Zheng et al. (2024). To minimize subjective bias, all measurements were performed independently by three trained researchers blinded to genotype identities, and the final VSG value for each plant was averaged from the three assessments.

Chlorophyll content in the ear leaves was measured spectrophotometrically according to Reis et al. (2019). Photosynthetic parameters, including Pn and Ci, were determined on ear leaves using a portable photosynthesis system (LI-6400, LI-COR) as described by Zuo et al (Xiuliang et al., 2015). Additionally, dry matter accumulation was calculated following the method of Fan et al. (2024).

#### 4.2.2 Chloroplast ultrastructure observation

Maize leaves sampled at a stage, b stage and c stage were cut into 1–2 mm sections and fixed in 2.5% glutaraldehyde. After rinsing with phosphate buffer, post-fixation in 1% osmium tetroxide, gradient ethanol dehydration, epoxy resin embedding, and ultrathin sectioning (70 nm), the sections were stained with uranyl acetate and lead citrate. Chloroplast structure, grana number, and osmiophilic granule changes were examined under a transmission electron microscope.

### 4.3 DNA library construction and BSA-seq analysis

#### 4.3.1 DNA library construction

Genomic DNA was extracted from leaves of T01, Mo17, and the F2:3 population. Based on phenotypic evaluation, 30 extreme stay-green and 30 extreme non-stay-green F2:3 individuals were selected, and their DNA were pooled in equal amounts to construct the stay-green pool and the non-stay-green pool, respectively. DNA from the parental lines was also extracted separately.

#### 4.3.2 BSA-seq analysis

Paired-end 150 bp resequencing was performed on the MGISEQ-2000 platform. After quality control of raw data, high-quality clean reads (Q30≥94.86%) were obtained and aligned to the maize B73 reference genome. SNP and InDel calling were carried out using GATK software. Following the method of Fan et al. (2025), we performed initial mapping by taking the intersection of results from seven algorithms, including DeepLearning, K value, ED4, ΔSNP-index, Smooth G, Smooth LOD, and Ridit. The significance threshold at the 95% confidence level was determined by 1000 permutation tests.

### 4.4 Transcriptome analysis

Based on phenotypic differences, leaves of T01 and Mo17 were sampled at stage a, stage b, and stage c, with three biological replicates per line per time point, totaling 18 samples. Total RNA was extracted using the TRIzol method, and RNA integrity was assessed with an Agilent 2100 bioanalyzer (RIN≥7). Strand-specific transcriptome libraries were constructed using the Illumina TruSeq Library Construction Kit and sequenced on the Illumina NovaSeq 6000 platform with 150 bp paired-end reads.

Raw reads were processed with fastp for quality control and adapter trimming to obtain clean reads. Clean reads were aligned to the maize B73 reference genome using HISAT2, and transcript assembly and quantification were performed with StringTie to calculate FPKM values.

Raw read counts were used as the input for DESeq2 to identify DEGs, and FPKM values were calculated for expression visualization. Differential expression analysis was conducted using the R package DESeq2, with significance thresholds set at |log_2_(fold change)|≥1 and adjusted P-value < 0.05. Functional annotation and enrichment analysis of differentially expressed genes (DEGs) were carried out using clusterProfiler for Gene Ontology (GO) terms, and KEGG pathway enrichment analysis was also performed, with P < 0.05 considered as the threshold for significant enrichment.

### 4.5 WGCNA

Genes with expression standard deviation ≤ 0.5 across all samples were excluded, and the remaining genes were used for co-expression network analysis using the Oebiotech online platform (https://cloud.oebiotech.com). The soft-thresholding power was determined according to the method described by Sánchez (Sánchez-Baizán et al., 2022), with β=30 selected based on the scale-free topology criterion. Module eigengenes were calculated and correlated with the VSG trait; modules with |r| > 0.8 and P < 0.01 were retained as significantly trait-associated modules. For each module, GS was defined as the correlation between gene expression and VSG, and kME as the correlation between gene expression and the module eigengene. The co-expression networks were visualized using Cytoscape (v3.10.0), and the top 10 genes ranked by kME within each module were selected as candidate hub genes (Zuo et al., 2018).

### 4.6 RT-qPCR validation

To verify the expression patterns of three candidate stay-green-related genes identified in this study, RT-qPCR was performed using the *ZmActin* gene as an internal reference, and relative expression levels were calculated using the 2^-ΔΔCt^ method (Dong et al., 2024). Expression validation was conducted in the stay-green line T01 and the non-stay-green line Mo17 at 10, 20, and 30 DAP, using the primer sequences listed in **Supplementary Table S11**. Each sample was assayed with three biological replicates and three technical replicates to ensure data reliability.

### 4.7 Statistical analysis

All experiments were performed with three biological replicates. Phenotypic evaluation used 3 plants per genotype per time point. Transcriptome sequencing and RT-qPCR each included three biological replicates per sample, with each replicate derived from an independent plant. Statistical analyses were performed using R software (version 4.1.0). For phenotypic comparisons between T01 and Mo17 at individual stages, Student’s t-test was used. To evaluate the effects of genotype, developmental stage, and their interaction on phenotypic and gene expression traits, two-way analysis of variance (ANOVA) was performed, followed by Tukey’s HSD test for multiple comparisons. DEGs were identified using DESeq2 with |log_2_(fold change)| ≥ 1 and adjusted *P* < 0.05. GO and KEGG enrichment were analyzed using clusterProfiler with *P* < 0.05 as significance threshold. Data are presented as mean ± SD.

## 5 Conclusion

In this study, we conducted an integrated analysis combining BSA-seq, RNA-seq, and WGCNA to systematically dissect the stay-green inbred line T01 and the non-stay-green line Mo17 in maize. BSA-seq mapped two candidate intervals associated with the stay-green trait on chromosomes 5 and 9, among which the interval on chromosome 5 represented a newly detected locus in T01. Temporal transcriptome analysis revealed that the maintenance of stay-green depends on stage-specific synergistic regulation across multiple pathways, including reactive oxygen species metabolism, photosynthetic light harvesting, and chloroplast structure maintenance. WGCNA identified six hub genes with high connectivity, with functions spanning transcriptional regulation, reactive oxygen species scavenging, and photosynthesis. By integrating BSA and WGCNA results, we identified three candidate genes, *ZmFSD3*, *ZmWRKY62*, and *ZmNAC29*, which were consistently detected by both genetic mapping and co-expression network analysis. Their differential expression patterns between T01 and Mo17 across developmental stages were further validated by RT-qPCR. These results suggest that these three genes may play potential roles in regulating the stay-green trait in maize. In conclusion, the stay-green trait in maize may be influenced by the regulation of WRKY and NAC transcription factors. Future studies employing gene editing or transgenic approaches should be conducted to further elucidate their functional roles. This study provides candidate genetic resources and a theoretical foundation for further investigation and molecular breeding of stay-green in maize.

## Data Availability

The data associated with this study have been deposited in NCBI SRA under BioProject accession number PRJNA1526346.All other data are available from the corresponding author upon reasonable request.
