# Supplementary material for: Integrated transcriptomic and BSA-seq analyses identify novel QTL and candidate genes for stay-green in maize
Source: Front Plant Sci. 2026 Sep 24;17:1936883. doi: 10.3389/fpls.2026.1936883 (PMC13645777; doi:10.3389/fpls.2026.1936883)
Supplement: Supplementary file 1 [file DataSheet1.docx]

Figure S1 Comparison of green correlation between 2 materials

Figure S2 Screening of soft-thresholding power for WGCNA

| RNA-seq | RT-qPCR |
| --- | --- |
| 114 | 96 |
| 1.55 | 1.45 |
| 1.7 | 2 |
| 1.1 | 1.58 |
| 3.7 | 4.25 |
| 2.8 | 2.55 |
| 1.8 | 1.95 |

Figure S3 Validation of RNA-seq expression by RT-qPCR for three candidate genes
