# Supplementary material for: Integrated transcriptomic and BSA-seq analyses identify novel QTL and candidate genes for stay-green in maize
Source: Front Plant Sci. 2026 Sep 24;17:1936883. doi: 10.3389/fpls.2026.1936883 (PMC13645777; doi:10.3389/fpls.2026.1936883)
Supplement: Supplementary file 2 [file Table1.docx]

Table S1 Morphometric parameters of chloroplast ultrastructure in figure 2

| Drawing number | Long axis of chloroplast/μm | Short axis of chloroplast/μm | Area of chloroplast/μm^2^ | Complete grana number/per μm^2^ |
| --- | --- | --- | --- | --- |
| A | 5.08±0.06 | 2.41±0.06 | 11.95±0.58 | 5±3 |
| B | 4.63±0.05 | 2.21±0.05 | 10.01±0.46 | 4±2 |
| C | 5.53±0.04 | 3.73±0.08 | 20.54±0.67 | 15±4 |
| D | 4.92±0.03 | 3.46±0.04 | 18.71±0.55 | 13±3 |
| E | 4.99±0.03 | 3.44±0.04 | 19.98±0.62 | 12±3 |
| F | 3.53±0.04 | 2.03±0.08 | 8.54±0.17 | 7±2 |

Table S2 Summary of sequencing and variant detection parameters for BSA-seq

| Parameter | Value |
| --- | --- |
| Sequencing depth | 30× |
| SNP/InDel density | 1.1 |
| Parental polymorphism rate | 1.11% |
| SNP-index distribution | 0.82 |

Table S3 Quality statistics of mapping with the reference genome

| Sample | Read sum | Base sum | Q30/% | GC content/% |
| --- | --- | --- | --- | --- |
| P_1_ | 489.3M | 73.3G | 95.19 | 49.17 |
| P_2_ | 488.0M | 73.2G | 94.86 | 47.81 |
| H-pool | 764.0M | 114.6G | 96.35 | 46.26 |
| L-pool | 781.0M | 117.1G | 96.24 | 45.22 |

Table S4 Statistics on the number of polymorphic markers on 10 chromosomes

| Chromosome | Total number of InDel markers | Number of polymorphic InDel markers |
| --- | --- | --- |
| Chr1 | 52 | 2 |
| Chr2 | 50 | 5 |
| Chr3 | 30 | 1 |
| Chr4 | 27 | 1 |
| Chr5 | 61 | 8 |
| Chr6 | 40 | 5 |
| Chr7 | 39 | 2 |
| Chr8 | 66 | 4 |
| Chr9 | 50 | 6 |
| Chr10 | 40 | 4 |
| Total | 455 | 38 |

Table S5 Statistical analysis of candidate intervals from 7 different algorithms

| Chromosome | Method | Start position/bp | Termination position/bp | Region size/Mb |
| --- | --- | --- | --- | --- |
| Chr5 | DeepLearning | - | - | - |
|  | K-value | - | - | - |
|  | ED4 | 54501987 | 57775143 | 3.27 |
|  | ΔSNP-index | 55730000 | **57093318** | 1.36 |
|  | Smooth G | 56018505 | 57857090 | 1.84 |
|  | Smooth LOD | 55541665 | 57726373 | 1.55 |
|  | Ridit | **56151304** | 57916845 | 1.77 |
| Chr9 | DeepLearning | - | - | - |
|  | K-value | 132325457 | **138243108** | 5.92 |
|  | ED4 | 136900892 | 150856319 | 13.96 |
|  | ΔSNP-index | **137052892** | 150747472 | 13.7 |
|  | Smooth G | 136872591 | 146249241 | 9.38 |
|  | Smooth LOD | 136901013 | 147447017 | 10.55 |
|  | Ridit | 136357422 | 147447235 | 10.39 |

Note: Values in bold indicate the final selected interval start and end positions.

Table S6 Function annotation of genes in Chr5

| Gene ID | Function | IP |
| --- | --- | --- |
| *Zm00001eb226510* | Hypothetical protein ZEAMMB73_Zm00001d014620 | https://maizegdb.org/gene_center/gene/Zm00001eb226510 |
| *Zm00001eb226520* | DNA-directed RNA polymerases II, IV and V subunit 8B | https://maizegdb.org/gene_center/gene/Zm00001eb226520 |
| *Zm00001eb226530* | AAA-ATPase | https://maizegdb.org/gene_center/gene/Zm00001eb226530 |
| *Zm00001eb226540* | Hypothetical protein ZEAMMB73_Zm00001d014624 | https://maizegdb.org/gene_center/gene/Zm00001eb226540 |
| *Zm00001eb226550* | AAA-ATPase | https://maizegdb.org/gene_center/gene/Zm00001eb226550 |
| *Zm00001eb226560* | U3 small nucleolar ribonucleoprotein IMP4 | https://maizegdb.org/gene_center/gene/Zm00001eb226560 |
| *Zm00001eb226570* | Homeobox-leucine zipper protein HOX18 | https://maizegdb.org/gene_center/gene/Zm00001eb226570 |
| *Zm00001eb226580* | Homeobox-leucine zipper protein HOX18 | https://maizegdb.org/gene_center/gene/Zm00001eb226580 |
| *Zm00001eb226590* | Homeobox-leucine zipper protein HOX18 | https://maizegdb.org/gene_center/gene/Zm00001eb226590 |
| *Zm00001eb226600* | Superoxide dismutase | https://maizegdb.org/gene_center/gene/Zm00001eb226600 |
| *Zm00001eb226610* | Hypothetical protein Zm00014a_024027 | https://maizegdb.org/gene_center/gene/Zm00001eb226610 |
| *Zm00001eb226630* | Hypothetical protein ZEAMMB73_Zm00001d030407 | https://maizegdb.org/gene_center/gene/Zm00001eb226630 |
| *Zm00001eb226640* | E3 ubiquitin-protein ligase BRE1-like 1 | https://maizegdb.org/gene_center/gene/Zm00001eb226640 |

Table S7 Function annotation of candidate genes in Chr9

| Gene ID | Function | IP |
| --- | --- | --- |
| *Zm00001eb395130* | Probable WRKY transcription factor 62 | https://maizegdb.org/gene_center/gene/Zm00001eb395130 |
| *Zm00001eb395140* | Lumenal binding protein cBiPe3 | https://maizegdb.org/gene_center/gene/Zm00001eb395140 |
| *Zm00001eb395150* | Lung seven transmembrane receptor family protein | https://maizegdb.org/gene_center/gene/Zm00001eb395150 |
| *Zm00001eb395160* | - | https://maizegdb.org/gene_center/gene/Zm00001eb395160 |
| *Zm00001eb395170* | - | https://maizegdb.org/gene_center/gene/Zm00001eb395170 |
| *Zm00001eb395180* | Uncharacterized protein LOC100304270 | https://maizegdb.org/gene_center/gene/Zm00001eb395180 |
| *Zm00001eb395190* | Hypothetical protein | https://maizegdb.org/gene_center/gene/Zm00001eb395190 |
| *Zm00001eb395200* | Hypothetical protein ZEAMMB73_Zm00001d047528 | https://maizegdb.org/gene_center/gene/Zm00001eb395200 |
| *Zm00001eb395210* | - | https://maizegdb.org/gene_center/gene/Zm00001eb395210 |
| *Zm00001eb395220* | Putative AGC-like protein kinase family protein isoform 1 | https://maizegdb.org/gene_center/gene/Zm00001eb395220 |
| *Zm00001eb395230* | Photosystem II repair protein PSB27-H1, chloroplastic | https://maizegdb.org/gene_center/gene/Zm00001eb395230 |
| *Zm00001eb395240* | Putative receptor-like protein kinase family protein | https://maizegdb.org/gene_center/gene/Zm00001eb395240 |
| *Zm00001eb395250* | Hypothetical protein Zm00014a_040220 | https://maizegdb.org/gene_center/gene/Zm00001eb395250 |
| *Zm00001eb395260* | Unknown | https://maizegdb.org/gene_center/gene/Zm00001eb395260 |
| *Zm00001eb395270* | Hypothetical protein ZEAMMB73_Zm00001d047536 | https://maizegdb.org/gene_center/gene/Zm00001eb395270 |
| *Zm00001eb395280* | Hypothetical protein Zm00014a_040217 | https://maizegdb.org/gene_center/gene/Zm00001eb395280 |
| *Zm00001eb395290* | Endo-1,4-beta-glucanase | https://maizegdb.org/gene_center/gene/Zm00001eb395290 |
| *Zm00001eb395300* | Putative RNA-binding zinc finger family protein | https://maizegdb.org/gene_center/gene/Zm00001eb395300 |
| *Zm00001eb395310* | Guanine nucleotide exchange factor SPIKE 1 isoform X3 | https://maizegdb.org/gene_center/gene/Zm00001eb395310 |
| *Zm00001eb395320* | NAC transcription factor 29 | https://maizegdb.org/gene_center/gene/Zm00001eb395320 |
| *Zm00001eb395330* | 17.6 kDa class II heat shock protein | https://maizegdb.org/gene_center/gene/Zm00001eb395330 |

Table S8 Transcriptome Sequencing Data Statistics

| Sample | clean-reads | mapped-reads | mapped-rate(%) |
| --- | --- | --- | --- |
| Ma1 | 42888832 | 35361841 | 82.45 |
| Ma2 | 47434418 | 37786257 | 79.66 |
| Ma3 | 40921186 | 34553849 | 84.44 |
| Mb1 | 49473398 | 41335024 | 83.55 |
| Mb2 | 44705950 | 35970407 | 80.46 |
| Mb3 | 45710004 | 37578194 | 82.21 |
| Mc1 | 49604668 | 37759073 | 76.12 |
| Mc2 | 58304954 | 42416854 | 72.75 |
| Mc3 | 49067544 | 36624014 | 74.64 |
| Ta1 | 44672922 | 36126992 | 80.87 |
| Ta2 | 46973396 | 38729565 | 82.45 |
| Ta3 | 45337266 | 35503613 | 78.31 |
| Tb1 | 32986302 | 23291624 | 70.61 |
| Tb2 | 45399694 | 34017990 | 74.93 |
| Tb3 | 42669454 | 31519925 | 73.87 |
| Tc1 | 46474978 | 32709089 | 70.38 |
| Tc2 | 43922438 | 36196481 | 82.41 |
| Tc3 | 47122680 | 33457102 | 71 |

Table S9 Distribution of number of genes in co-expression modules

| Modules | The number of genes |
| --- | --- |
| brown 2 | 2363 |
| brown | 2021 |
| antiquewhite 2 | 1408 |
| darkolivegreen 1 | 1349 |
| coral 3 | 1211 |
| blueviolet | 1075 |
| mediumpurple 1 | 266 |
| coral | 210 |
| skyblue | 186 |
| darkseagreen 1 | 179 |
| coral 1 | 153 |
| palevioletred 3 | 151 |
| darkslateblue | 131 |
| blue 4 | 122 |
| pink 4 | 89 |
| mediumpurple 4 | 79 |
| lightcoral | 76 |
| mediumpurple 3 | 70 |
| chocolate 3 | 57 |
| plum | 52 |
| firebrick 4 | 48 |
| darkseagreen 3 | 43 |
| deeppink | 37 |

Table S10 WGCNA candidate genes

| Moudle | Genes ID | Functional annotation | Correlation strength |
| --- | --- | --- | --- |
| darkoolivegreen 1 | *Zm00001eb057820* | WRKY transcription factor 57 | 0.949 |
|  | *Zm00001eb106810* | Chlorophyll a-b binding protein CP29.1 chloroplastic | 0.903 |
|  | *Zm00001eb226600** | Superoxide dismutase | 0.987 |
|  | *Zm00001eb358500* | Uroporphyrinogen decarboxylase 1, chloroplastic | 0.989 |
| brown 2 | *Zm00001eb395130** | WRKY transcription factor 62 | -0.993 |
|  | *Zm00001eb395320** | NAC transcription factor 29 | -0.992 |

Note: * indicates the genes co-mined for QTL and WGCNA.

Table 11 Primers for RT-qPCR validation of the candidate genes.

| Gene name | Gene ID | Sense primer | Anti-Sense primer |
| --- | --- | --- | --- |
| *ZmFSD3* | *Zm00001eb226600* | CTGGGAGCGGCAAGAAAATAC | TGATGGTGCTTACCCCAGTG |
| *ZmWRKY62* | *Zm00001eb395130* | TCCACGTACGTCGTCACCTA | TTGTTGGAGTTGGAGCCGAA |
| *ZmNAC29* | *Zm00001eb395320* | AAATGATGCCTTTTGCTTGCC | CAAACCGATGCGATCTTGCG |
| *ZmActin* | *GRMZM2G126010* | TACGAGATGCCTGATGGTCAGGTCA | TGGAGTTGTACGTGGCCTCATGGAC |
